# Environmental Risk Factors for Parkinson's Disease: A Critical Review and Policy Implications

**DOI:** 10.1002/mds.30067

**Published:** 2024-11-27

**Authors:** Kajsa Atterling Brolin, Eva Schaeffer, Ashvin Kuri, Isabell Katharina Rumrich, Artur Francisco Schumacher Schuh, Sirwan K.L. Darweesh, Valtteri Kaasinen, Anna‐Maija Tolppanen, Lana M. Chahine, Alastair J. Noyce

**Affiliations:** ^1^ Translational Neurogenetics Unit, Department of Experimental Medical Science Lund University Lund Sweden; ^2^ Centre for Preventive Neurology, Wolfson Institute of Population Health Queen Mary University of London London UK; ^3^ Department of Neurology, University Hospital Schleswig‐Holstein Campus Kiel and Kiel University Kiel Germany; ^4^ School of Pharmacy University of Eastern Finland Finland; ^5^ Department of Health Protection Finnish Institute for Health and Welfare Finland; ^6^ Departamento de Farmacologia Universidade Federal do Rio Grande do Sul Porto Alegre Brazil; ^7^ Serviço de Neurologia Hospital de Clínicas de Porto Alegre Porto Alegre Brazil; ^8^ Radboud University Medical Center Donders Institute for Brain, Cognition and Behaviour, Department of Neurology, Center of Expertise for Parkinson & Movement Disorders Nijmegen The Netherlands; ^9^ Clinical Neurosciences University of Turku Turku Finland; ^10^ Neurocenter Turku University Hospital Turku Finland; ^11^ Department of Neurology University of Pittsburgh Pittsburgh Pennsylvania USA

**Keywords:** Parkinson's disease, environmental factors, pesticides, air pollution, trichloroethylene

## Abstract

The age‐standardized prevalence of Parkinson's disease (PD) has increased substantially over the years and is expected to increase further. This emphasizes the need to identify modifiable risk factors of PD, which could form a logical entry point for the prevention of PD. The World Health Organization (WHO) has recommended reducing exposure to specific environmental factors that have been reported to be associated with PD, in particular pesticides, trichloroethylene (TCE), and air pollution. In this review we critically evaluate the epidemiological and biological evidence on the associations of these factors with PD and review evidence on whether these putative associations are causal. We conclude that when considered in isolation, it is difficult to determine whether these associations are causal, in large part because of the decades‐long lag between relevant exposures and the incidence of manifest PD. However, when considered in tandem with evidence from complementary research lines (such as animal models), it is increasingly likely that these associations reflect harmful causal effects. Fundamentally, whilst we highlight some evidence gaps that require further attention, we believe the current evidence base is sufficiently strong enough to support our call for stronger policy action. © 2024 The Author(s). *Movement Disorders* published by Wiley Periodicals LLC on behalf of International Parkinson and Movement Disorder Society.

Parkinson's disease (PD) has been reported to be the fastest growing neurological disorder worldwide in prevalence and deaths.^1^, ^2^ The age‐standardized increase in prevalence was 60% between 1990 and 2021, leading to ~11.8 million PD cases globally and highlighting the importance of identifying modifiable risk factors of PD.^2^ In 2022, the World Health Organization (WHO) published a technical brief outlining the global burden, treatment gaps, and crucial areas for action for PD.^3^ This report provided considerations for policy implementation and research, and included key actions for prevention and risk reduction to lessen the anticipated increase in the global burden of PD. The WHO technical brief, which was used in this review to narrow the focus on modifiable factors linked to PD, recommended reducing exposure to environmental factors associated with PD risk, including the need to “ban pesticides (eg, paraquat and chlorpyrifos) and chemicals (eg, trichloroethylene)” and to “accelerate action to reduce levels of and exposure to air pollution…”.

This review (for details of the literature search see Box 1) examines the evidence linking these modifiable factors (pesticides, trichloroethylene [TCE] and air pollution) to PD to support actions aimed at understanding and addressing them through public education, policy, and legislative changes. In order to convey available evidence, we provide a visual summary categorized according to the Bradford Hill (BH) criteria. We also aim to critically evaluate the epidemiological and pathophysiological research findings for these factors and make recommendations for policy change and further evidence synthesis.

Box 1 **Methodology/literature search**

This narrative review was undertaken following a literature search on the MEDLINE database using PubMed to identify articles published from 1969 to April 2024. Articles were assessed for their relevance to the subject matter and the search was restricted to articles written in the English language. The following keywords were used individually or/and in combination with “Parkinson's disease”: environmental risk factor(s), gene–environment, pesticide(s), rotenone, paraquat, maneb, organophosphate(s), organochlorine(s), solvents, trichlorethylene, TCE, herbicide(s), insecticide(s), fungicide(s), pyrethroids, glyphosate, chlorpyrifos, dieldrin, terbufos, lindane, simazine, atrazine, air pollution, nitrogen, particle(s), and particulate. Also published meta‐analytical data in relation to these specific risk factors were collected (Tables 1 and 2).

## Pesticides

‘Pesticides’ comprise a variety of compounds that regulate unwanted organisms, particularly in agriculture, and include insecticides, fungicides, and herbicides. Exposure to pesticides via inhalation or skin contact represents a specific occupational risk, particularly for those working in agriculture. However, non‐occupational exposure also occurs via multiple routes, for example, intake of contaminated water or food and accidental ingestion/contact with pesticide‐contaminated environmental media.^4^, ^5^, ^6^ Household exposure can be significant, especially in countries where household insecticides are used to prevent mosquito‐borne diseases.^7^ Pesticide exposure has been linked to an increased risk of neurodegenerative diseases including amyotrophic lateral sclerosis (ALS)^8^ and Alzheimer's disease (AD),^9^ and there are numerous lines of evidence supporting a link between pesticide exposure and PD, both from case–control and cohort studies (Table 1). However, as for all environmental exposures, proving a causal link is challenging. Whilst some studies treat pesticides as a homogenous group of chemicals, considering pesticides individually is necessary. Other environmental exposures co‐occurring with pesticide use (ie, confounding) and/or genetic susceptibility could contribute to observed associations between pesticides and PD. Additionally, information bias and inaccuracies in exposure assessment may contribute to the observed association. Nevertheless, considering that the average total global pesticide use increased in the most recent decade by nearly 50% compared with the 1990s,^10^ the potential link to PD must be clearly understood.

**TABLE 1:** Available meta‐analyses of the association between pesticides and Parkinson's disease

		Meta‐analysis (reference)														
		Chambers‐Richards et al. 2021. PMID: 34796708	Gunnarsson et al. 2019. PMID: 30691095	Vaccari et al. 2019. PMID: 31476981	Tangamornsuksan et al. 2019. PMID: 30474499	Yan et al. 2018. PMID: 29729297	Ahmed et al. 2017. PMID: 28412655	Gunnarsson et al. 2017. PMID: 28379585	Breckenridge et al. 2016. PMID: 27055126	Pezzoli et al. 2013. PMID: 23713084	van der Mark 2012. PMID: 22389202	Noyce et al. 2012. PMID: 23071076	Van Maele‐Fabry et al. 2012 PMID: 22698719	Van der Mark et al. 2012. PMID: 22389202	Priyadarshi et al. 2001. PMID: 11437458	Priyadarshi et al. 2000. PMID: 11022853
Reporting RR or OR		RR	RR	OR	OR	OR	OR	RR	RR	OR	RR	RR	RR	RR	OR	OR
Pesticide																
General	Studies in meta‐analysis (n)	11	24			10	64	23	49	51	46	38	12	39	14	19
	OR/RR (95% CI)	**1.41 (1.20–1.65)**	**1.66 (1.42–1.94)**			**1.11 (1.05–1.18)** *	**1.46 (1.21‐1.77)**	**1.67 (1.42–1.97)**	**1.22 (1.18–1.27)**	**1.76 (1.56–2.04)**	**1.62 (1.40–1.88)**	**1.78 (1.50–2.10)**	**1.28 (1.03–1.59)**	**1.62 (1.40–1.88)**	**1.85 (1.31–2.60)**	**1.94 (1.49–2.53)**
Insecticides	n								17	18	14					
	OR/RR (95% CI)								**1.32 (1.14–1.52)**	**1.53 (1.12–2.08)**	**1.50 (1.07–2.11)**					
Herbicides	n								18	19	14					
	OR/RR (95% CI)								**1.20 (1.06–1.36)**	**1.33 (1.08–1.65)**	**1.40 (1.08–1.81)**					
Fungicides	n								10	12						
	OR/RR (95% CI)								0.94 (0.75–1.19)	0.97 (0.69–1.38)						
Paraquat	n			11	13				13	7						
	OR/RR (95% CI)			**1.24 (1.03–1.49)**	**1.64 (1.27–2.13)**				**1.47 (1.01–2.13)**	**2.19 (1.48–3.26)**						
Maneb	n									4						
	OR/RR (95% CI)									1.49 (0.85–2.63)						

Significant associations are highlighted in bold.

* Ten‐year exposure duration.

No meta‐analyses were available for other pesticides highlighted in the review, namely rotenone, glyphosate, pyrethroids, chlorpyrifos, and terbufos.

Abbreviations: RR, risk ratio; OR, odds ratio; CI, confidence interval.

### Current Evidence for a Causal Link

The relationship between pesticide exposure and PD gained attention following the discovery in 1983 that 1‐methyl‐4‐phenyl‐1,2,3,6‐tetrahydropyridine (MPTP), a substance structurally similar to the pesticide paraquat, resulted in rapid‐onset parkinsonism following intravenous injection.^11^ Since then, numerous epidemiological studies and meta‐analyses have reported associations between PD and pesticide exposure (Table 1), as well as with factors putatively related to pesticide exposure (ie, rural living, farming, and well‐water drinking).^12^, ^13^, ^14^, ^15^

Evidence of a possible dose–response relationship between overall pesticide exposure and PD has been reported with a 5% versus 11% increased risk for 5 and 10 years of exposure duration, respectively,^16^ and one prospective study reported increased mortality in PD patients with occupational exposure to pesticides.^17^ A recent study identified an association with PD with long‐term exposure to 53 different pesticides (25 at a false discovery rate [FDR] of ≤0.01),^18^ whereas residential and workplace proximity to higher amounts of 10 of these 53 pesticides was associated with a faster PD symptom progression.^19^, ^20^

In 2016, a systematic review funded by Syngenta Crop Protection, LLC was published, in which they used the BH framework to investigate the association between PD and factors such as pesticides.^15^ It should be highlighted that Syngenta manufactures a significant proportion of commercially sold paraquat (a quarter of global paraquat sales according to its website). In this work, the authors observed a consistent positive association between pesticide use and PD, with an estimated relative risk of 1.56 (95% Cl = 1.37–1.77). However, they reported a lack of specificity and biological plausibility and concluded that there was insufficient evidence for a causal relationship with PD.^15^

### Paraquat

In 1987, a small ecological study observed that the differences in regional prevalence of PD were linked to soil and water contamination with agricultural pesticides.^21^ The herbicide paraquat was one of the most frequently used. In general, available evidence of an association between PD and specific pesticides remains sparse and/or uncertain. However, the association between paraquat and PD has been consistently reported (Table 1). One meta‐analysis from 2019 of 1244 paraquat‐exposed versus 5026 non‐exposed individuals reported a 43% higher odds of PD (odds ratio [OR] = 1.43, 95% CI = 1.06–1.91, *P* = 0.02, I2 = 56%).^22^ Only three^12^, ^23^, ^24^ of the 10 included case–control studies were at low risk of bias, in particular with respect to confidence in the assessment of previous paraquat exposure.^22^ These three studies reported details for paraquat exposure (ie, frequency and duration) through job and crop exposure matrices^12^, ^23^ or through estimated exposure by georeferencing coding systems,^24^ whereas most other studies collected exposure information through interviews/questionnaires. A recent publication from the Parkinson Environment Gene (PEG) study further supports an association between PD and paraquat in which paraquat exposure was based on historical pesticide application records. Living near and workplace proximity to commercial applications were associated with an approximate doubling of odds, and an annual paraquat exposure intensity of ~4.5 kilograms applied per acre/year was associated with an increased PD risk (OR = 2.08, 95% CI = 1.31–3.38).^25^

Despite consistent evidence from several epidemiological studies, reports from the Agricultural Health Study (AHS) showed a lack of an association between paraquat and PD (hazard ratio [HR] = 1.09, 95% CI = 0.84–1.41).^26^, ^27^, ^28^ In the AHS prospective cohort, 8613 private pesticide applicators and their spouses had self‐reported paraquat use, of which 87 (1%) had developed PD at the 20‐year follow‐up.^27^ The absence of an association between paraquat and PD in the AHS is at odds with the magnitude of the positive association between paraquat and PD in case–control studies.

**FIG. 1:**
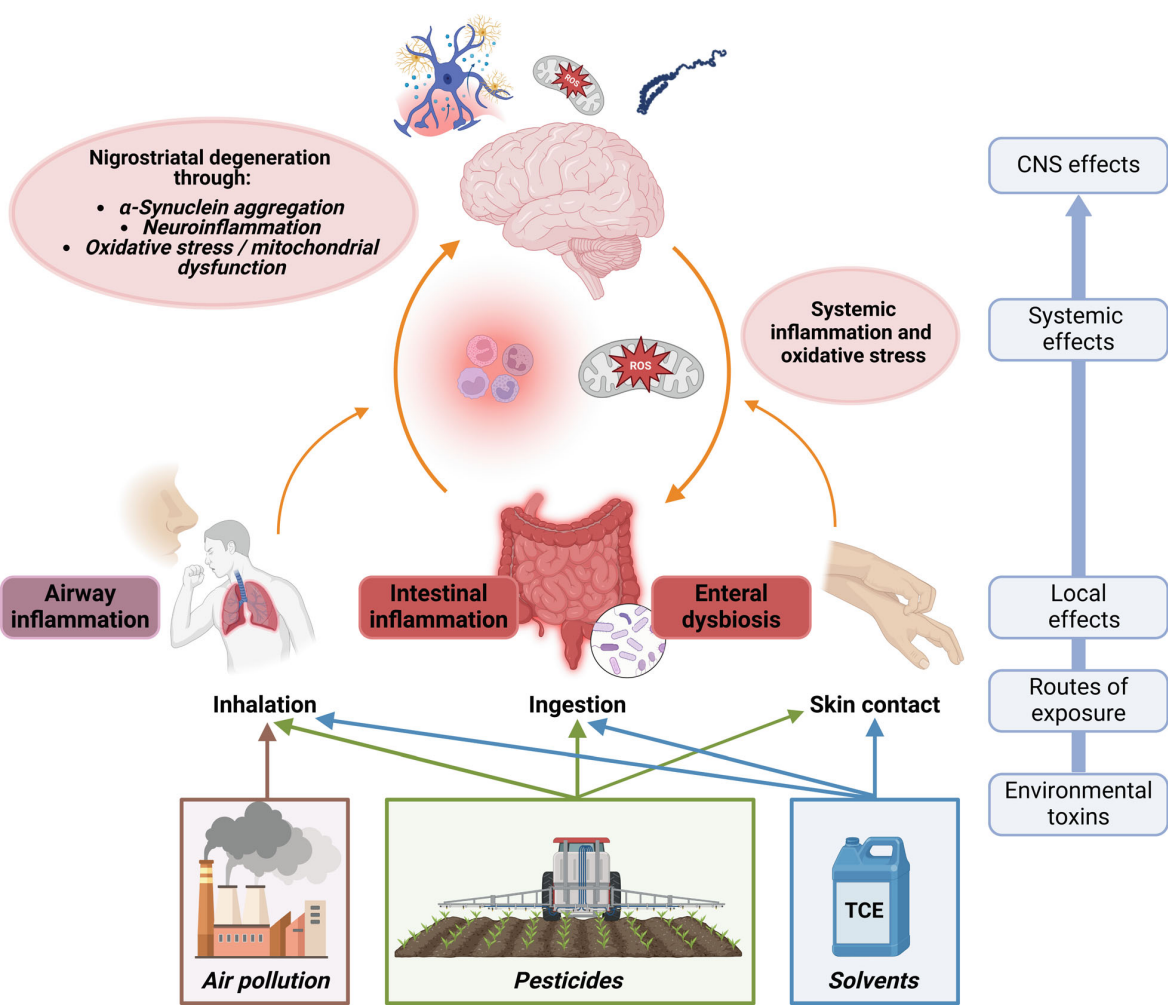
Environmental toxicants can have an effect on humans through various exposure routes and initially lead to local reactions. Abbreviations: CNS, Central Nervous System; TCE, trichloroethylene. Created with BioRender.com. [Color figure can be viewed at wileyonlinelibrary.com]

In addition to the loss of dopaminergic cells in the substantia nigra (SN) and abnormal accumulation of proteins (ie, α‐synuclein [α‐syn]) within neurons (Lewy bodies and Lewy neurites), mitochondrial dysfunction, oxidative stress, and neuroinflammation are believed to play important roles in PD pathology.^29^ The key pathophysiological mechanisms by which pesticides (and TCE and certain air pollutants) may trigger PD pathology appear to mainly be through mitochondrial dysfunction, oxidative stress, and neuroinflammation (Fig. 1). Exposure to paraquat causes selective dopaminergic toxicity in the SN of rodents.^30^, ^31^ Paraquat can cross the blood–brain barrier (BBB),^32^, ^33^ and acts as a redox cycling compound, inducing the formation of reactive oxygen species (ROS), thus leading to highly increased mitochondrial and cytosolic oxidative stress.^34^, ^35^ The resulting oxygen species interact with additional inhibitory effects of paraquat on proteasome function and autophagy, and the induction of apoptotic pathways through elevated cytochrome release.^36^ Of relevance for human exposure routes, animal studies of prolonged low‐dose inhalation of paraquat revealed that paraquat inhalation resulted in accumulation in various brain regions with the highest concentration in the olfactory bulb and caused persistent deficits in olfactory discrimination in male mice.^37^ Additionally, paraquat may directly promote the formation of α‐syn aggregation and fibril formation.^38^, ^39^, ^40^

### Maneb

Studies have reported a synergistic effect on PD risk for occupational co‐exposure to paraquat and dithiocarbamates (ie, the fungicide maneb).^22^, ^41^ Maneb exposure separately has been linked to risk of PD.^41^ However, this study only included a few “maneb‐only”‐exposed individuals and it has not been confirmed in other studies that maneb exposure alone is associated with PD risk.^27^, ^42^ Possible synergistic effects of paraquat and maneb have been observed in animal studies, with maneb affecting the mitochondrial complex III,^43^ but the combination of both potentiated effects on nigrostriatal dopaminergic neurons.^44^, ^45^

### Rotenone

Rotenone, which is used for animal models of PD,^46^ has only been seen to be linked to PD in a few case–control studies (any previous use of rotenone: OR = 2.5, 95% CI = 1.3–4.7^12^ and OR = 10.0, 95% CI = 2.9–34.3).^47^ However, the potential of rotenone to induce α‐syn aggregation followed by nigrostriatal degeneration of dopaminergic neurons has been intensively investigated in rodents.^48^, ^49^, ^50^, ^51^, ^52^, ^53^, ^54^ A recent study showed that rotenone‐exposed rats displayed transient motor slowing during exposure, but progressive motor abnormalities did not appear until 3 months after exposure, with the highest α‐syn accumulation after 9 months.^46^ Rotenone can cross the BBB^55^, ^56^ and similar to MPP+ (the active toxic compound of MPTP) rotenone accumulates in mitochondria and acts on the mitochondrial complex I, resulting in increased ROS levels.^57^, ^58^, ^59^ Moreover, rotenone appears to induce several other neurotoxic mechanisms, including proteasome inhibition and microtubule destabilization.^51^, ^60^, ^61^, ^62^ Additionally, rotenone (and paraquat) exposure can induce pathology in the gastrointestinal system in rodents, including enteral α‐syn aggregation, production of pro‐inflammatory cytokines in enteral glial cells, and a retrograde propagation of α‐syn pathology via the vagus nerve.^63^, ^64^, ^65^

### Glyphosate and Pyrethroids

The herbicide glyphosate, which was debated when the European Union (EU) was renewing its marketing authorization, among other reasons due to its potential link to PD,^66^ has only been reported in one epidemiological study to be associated with PD.^67^ Considering its wide use in agriculture, the potential risk of this pesticide needs to be further evaluated. This is also the case for commonly used household pesticides (ie, pyrethroids) as it has been observed that individuals with high exposure to household pesticides have a two times higher PD risk.^7^ For both glyphosate and pyrethroids, evidence of oxidative stress and dopaminergic uptake have been reported in animal models.^68^, ^69^, ^70^, ^71^, ^72^

### Organochlorine

In a review summarizing organochlorine pesticides as risk factors for PD, the organochlorines dieldrin and β‐hexachlorocyclohexane (HCH) were implicated. A total of nine studies were included, all small and reporting ORs between 1.1 and 5.8.^73^ In the AHS study of 38,274 pesticide applicators and 27,836 spouses, exposure to three organochlorine insecticides (chlordane, dichlorodiphenyltrichloroethane (DDT), and toxaphene) was associated with PD, but only among individuals reporting a history of head injury.^27^ Other small studies support a link between organochlorines and PD.^74^, ^75^ Some have investigated the neurotoxic properties of organochlorines, in particular dieldrin in *in vitro* and few *in vivo* studies where dieldrin induced for example, dopaminergic cell death, oxidative stress, and inflammation.^76^, ^77^, ^78^, ^79^, ^80^ The effects on the nigrostriatal system have not been reproduced in all animal studies,^81^, ^82^ but the accumulation of dieldrin in the brain has been linked to PD in small autopsy studies.^83^, ^84^ Animal models investigating dieldrin have also shown that developmental dieldrin exposure results in increased susceptibility to PD‐like pathology including sex‐specific poised epigenetic states, which may mediate sensitivity to subsequent toxic stimuli and contribute to the development of late‐life neurodegenerative disease.^85^, ^86^, ^87^, ^88^

### Organophosphates

Exposure to chlorpyrifos, a widely used organophosphate, has been associated with up to a two‐fold increased PD odds in two case–control studies (OR = 2.0, 95% CI = 1.02–3.8^47^ and OR = 1.56, 95% CI = 1.02–2.40^89^). Chlorpyrifos acts primarily as an acetylcholinesterase inhibitor and has various effects on the fetal and adult nervous systems.^90^ Prenatal chlorpyrifos exposure has been seen to be linked to structural brain abnormalities and potentially slowed brain growth *in utero*.^91^ In addition to the acute symptoms of cholinergic toxicity, acute parkinsonism following organophosphate poisoning has been reported in several case reports.^92^, ^93^, ^94^, ^95^, ^96^, ^97^ Of relevance to neurodegenerative diseases are the promotion of oxidative stress^98^, ^99^, ^100^ and neuroinflammation,^101^, ^102^ in both cell and animal models. Some rodent studies showed degeneration of the dopaminergic system after exposure to chlorpyrifos.^103^, ^104^, ^105^ Evidence of synergistic neurotoxicity and increased striatal α‐syn accumulation *in vivo* was seen with a combination of chlorpyrifos and paraquat.^106^ Additionally, organophosphates have been linked to PD in case–control studies.^107^, ^108^, ^109^, ^110^ However, a report from the AHS cohort, evaluating 16,843 chlorpyrifos‐exposed individuals, revealed no association between chlorpyrifos and PD (OR = 0.92, 95% CI = 0.74–1.13).^26^

### Other Pesticides

Exposure to other pesticides, namely the insecticide terbufos (HR = 1.31, 95% CI = 1.02–1.68) and the herbicide trifluralin (HR = 1.29, 95% CI = 0.99–1.70), were associated with PD in the AHS.^27^ Little previous research has been published regarding terbufos and trifluralin and PD. Terbufos has been associated with dream‐enactment behaviors in the AHS cohort, a characteristic feature of rapid eye movement sleep behaviour disorder (RBD).^111^ Only a small study of 69 patients with PD and 237 controls has previously been published showing that trifluralin was associated with PD among those with occupational exposure (OR = 5.5, 95% CI = 1.1–27.1).^112^ The effects of trifluralin on midbrain dopaminergic neurons derived from pluripotent stem cells was recently investigated, demonstrating that trifluralin induces dopaminergic cell death by inducing mitochondrial dysfunction.^18^ Associations between PD and the pesticides lindane, simazine, and atrazine have been explored in a large, population‐based study of 21,549,400 Medicare beneficiaries (>67 years) using pesticide data obtained from the US Geological Survey.^113^ Atrazine is a widely used herbicide with neurotoxic effects on dopaminergic neurons in vitro^114^, ^115^ and in vivo.^116^, ^117^

Further supporting the link between pesticide exposure and PD come from reports of gene–environment interactions (GxE) between pesticides and PD,^118^, ^119^ including a synergistic effect and further increased PD risk following pesticide exposure and carrying variants in genes, namely *BCHE* in a study of 416 PD patients and 445 controls with self‐reported pesticide exposure.^120^ Multiple GxE studies have additionally been conducted in the PEG study,^20^ showing interactions between pesticide exposure (or pesticide‐related exposure) and genes (ie, *HLA‐DRA*,^121^ *ALDH2*,^122^, ^123^, ^124^, ^125^ *ABCB1*,^126^ *NFE2L2*, and *PPARGC1α* ^127^) in PD. Results from the PEG study have also suggested that altered lysosomal function may underlie PD susceptibility in individuals exposed to pesticides as enrichment of variants in lysosomal pathways has been seen in PD patients exposed to agricultural pesticides.^128^ Other studies have also reported GxE relevant to PD, supporting a potential interaction between organochlorines and the gene *ABCB1*.^129^, ^130^

To investigate causal relationships in epidemiology, use of the BH criteria is a well‐recognized approach. However, the BH criteria were never designed to be an exhaustive checklist, and absolute fulfilment of all criteria is not a prerequisite for causation to be concluded. Here, we use the criteria to help assess whether an association between the variable of interest (eg, pesticides) and the outcome (PD) is likely to be causal rather than merely correlated (Table 3). In general, the evidence for a relationship between pesticide exposure and PD according to the BH criteria is clear. Multiple epidemiological studies exist, both case–control and cohort studies, along with animal studies that support a link between pesticides and PD. However, there is a lack of studies with supporting evidence for criteria relating to, for example, specificity, temporality, and biological gradient for many specific pesticides (ie, maneb and glyphosate).

## Trichlorethylene (TCE)

Trichlorethylene (TCE) has been a widely used solvent since the 1920s and is used in many industrial operations. TCE is a volatile and water‐soluble compound, released into the environment, and can be found in drinking water and soil, as well as in various human fluids, including urine, blood, or breast milk. All routes of exposure, including inhalation, ingestion, or via the skin, have been linked to an increased risk of various types of cancer, including kidney, liver, and non‐Hodgkin lymphoma.^131^ The carcinogenic properties of TCE are through its metabolites. The main metabolic pathways for TCE are cytochrome P450 (CYP)‐dependent oxidation and glutathione (GSH) conjugation by GSH S‐transferases (GSTs). The CYP pathway primarily yields chemically stable end products, but the metabolite of the GST pathway is further processed into highly reactive species that are known to be mutagenic. Specific TCE metabolites formed through CYP‐dependent oxidation may, however, also be genotoxic and mutagenic.^132^ Enzymes involved in TCE metabolism, particularly CYP enzymes, are highly variable across sexes, tissues, and individuals^133^, ^134^, ^135^ and variability in CYP activity, for example, due to genetic variability, can influence CYP‐dependent metabolism of TCE, which could alter the balance between the CYP and the GST pathways.^132^ Associations with TCE have been described with various other diseases including PD.^131^, ^136^, ^137^ TCE can cross the BBB and accumulate in tissues and body fluids.^138^ Initial reports on the potential neurotoxicity of TCE and its metabolites were published decades ago, but compared with the more extensive evidence surrounding pesticide exposure, epidemiological and animal studies regarding TCE are still limited.

The potential link between PD and TCE has gained more attention and evidence in recent years with a few case studies,^139^, ^140^, ^141^, ^142^, ^143^ and a small twin study in 2012 showed that TCE exposure was linked to a higher risk of PD (OR = 6.1; 95% CI = 1.2–33).^144^ More recently, compelling evidence of a link between volatile solvents (including TCE) and PD was published from a cohort study of >340,000 American military service members showing a 70% (OR = 1.70, 95% CI = 1.39–2.07) higher risk of PD among veterans stationed at a camp with water contaminated with volatile solvents during 1975–1985.^145^ PD diagnosis was based on medical record review (not clinical evaluation) and associations were attenuated when restricted to cases ascertained before 2017 (OR = 1.28, 95% CI = 1.00–1.64). This was the year the US Congress and Veterans Administration designated PD a presumptive service‐connected condition for veterans who served at Camp Lejeune from 1953 to 1987, making them eligible for benefits.^145^

Animal studies demonstrate that after oral administration of TCE there is a selective and dose‐dependent loss of dopaminergic neurons, as well as α‐syn inclusions in the SN and dorsal motor nucleus of the vagus nerve.^140^, ^141^, ^146^, ^147^, ^148^, ^149^, ^150^ Additionally, rats exposed to chronic TCE inhalation had degradation of nigrostriatal dopaminergic neurons, α‐syn accumulation in dopaminergic neurons, and showed motor and gait impairments.^150^ Similar to rotenone, the main effects of TCE could be attributed to a reduction in mitochondrial complex I enzyme activity.^141^, ^146^, ^147^, ^151^ Of relevance to potential GxE is the finding of increased LRRK2‐activity in the nigrostriatal tract of orally TCE‐exposed rats and an impairment of endolysosomal function.^148^

The available literature regarding an association between TCE and PD is lacking evidence for specificity and dose–response but shows consistency in other categories (ie, experiment, strength, consistency, and plausibility) (Table 3). Considering the large number of individuals worldwide that have been and still are being exposed to TCE, further studies are needed to understand the potential hazardous effects of TCE and its link to PD. However, monitoring systems for detecting TCE contamination in developing countries are sparse.

## Air Pollution

Ambient air pollution is a mixture of gaseous and particulate matter that has been associated with various adverse health effects (cardiovascular, pulmonary, neurological) and excess mortality. Particulate matter (PM) air pollution consists of different sized particles (eg, PM10 with a diameter of <10 μm, PM2.5 < 2.5 μm, and ultrafine particles <0.1 μm). The composition of particles differs as they are aggregated from several components (eg, black and organic carbon molecules, mineral dust, and secondary organic aerosols). Some of these components are characteristic of specific sources, such as black carbon for combustion processes and ammonium for agriculture. Nitrogen dioxide (NO_2_) is a gaseous marker for traffic‐related pollution, whereas secondary organic aerosols are related to long‐range transport. Organic carbons in the air pollution mixture may consist of known harmful or carcinogenic molecules such as polycyclic aromatic hydrocarbons (PHAs), iron, and other metals that are constituents of particles. Additionally, transition metals and lipopolysaccharides may be adsorbed onto particles. As the concentrations of pollutants and particle components differ across space and time, the mixture differs between studies, and even study participants. The effects of air pollution on the central nervous system have been investigated, focusing on cerebrovascular disease, AD, and PD.^152^, ^153^, ^154^, ^155^, ^156^ Oxidative stress and inflammation have been identified as key pathophysiological mechanisms linking neurological diseases to air pollution.^153^, ^156^, ^157^, ^158^

Some studies have provided evidence for associations between air pollution exposure and PD. For example, indications of a possible association have been found for PM2.5 and ozone (O_3_), but the available evidence for an association between specific air pollutants and PD remains unclear, with several meta‐analyses reaching null conclusions (Table 2). Only one meta‐analysis published in 2019 reported a clear association between PM2.5 exposure and increased PD risk (OR = 1.34, 95% CI = 1.04–1.73).^159^ However, the reliability was questionable due to erroneously high‐risk estimates (ie, higher than reported in the original publication) included in this meta‐analysis for at least two individual studies.^160^, ^161^ An updated systematic literature review and meta‐analysis is underway and will include an assessment of the exposure–response function, increasing the available evidence.^162^

**TABLE 2:** Available meta‐analysis of the association between specific air pollutants and Parkinson's disease

Air pollutant		Meta‐analysis (reference)						
		Wang et al. 2020. PMID: 31894453	Gong et al. 2023. PMID: 36763275	Dhiman et al. 2023. PMID: 35262433	Kasdagli et al. 2019. PMID: 30606679	Hu et al. 2019. PMID: 30391837	Han et al. 2020. PMID: 31770719	Fu et al. 2019. PMID: 30577116
PM2.5	Studies in meta‐analysis (n)	6	8	6	8	6	9	7
	OR (95% CI)	1.21 (0.95–1.54)	**1.17 (1.00–1.33)**	**1.01 (1.00–1.02)**	1.06 (0.99–1.14)	1.21 (0.95–1.54)	1.08 (0.98–1.19)	**1.34 (1.04–1.73)**
PM10	n	4	4		7	5	6	
	OR (95% CI)	1.01 (0.97–1.05)	1.00 (0.98–1.01)		0.99 (0.96–1.01)	1.00 (0.98–1.02)	0.99 (0.97–1.01)	
NO_2_	n			6	8		8	
	OR (95% CI)			**1.01 (1.00–1.02)** ^a^	1.01 (0.98–1.03)		1.03 (0.99–1.07)	
O_3_	n			4	5		5	
	OR (95% CI)			**1.01 (1.00–1.02)** ^b^	**1.01 (1.00–1.02)** ^c^		**1.01 (1.00–1.02)**	
CO	n			4	3		3	
	OR (95% CI)			1.64 (0.96–2.78)^d^	1.34 (0.85–2.10)^e^		1.32 (0.82–2.11)^e^	
Pmcoarse	n				4	2		
	OR (95% CI)				0.97 (0.93–1.01)	0.99 (0.96–1.01)		
NO_x_	n				5		4	
	OR (95% CI)				1.00 (0.98–1.03)		1.00 (0.98–1.03)	
SO_2_	n				3			
	OR (95% CI)				0.98 (0.79–1.21)^f^			

Significant associations are highlighted in bold.

^a^ Per 1 μg/m^3^.

^b^ Per 1 ppb (parts per billion).

^c^ Per 5 ppb.

^d^ Per 1 ppm (parts per million).

^e^ Per 1 mg/m^3^.

^f^ High vs. low.

ORs are per 10 μg/m^3^ increment of air pollutant, unless indicated otherwise.

Abbreviations: PM, particulate matter; OR, odds ratio; CI, confidence interval.

One Korean cohort study of >313,000 participants observed an association between PM2.5 exposure and PD, which was limited to those aged >65 years, male, or living in Metropolitan cities.^163^ A second Korean cohort study of >1 million participants (partly overlapping with the previous study) reported no association between PM2.5 and PD. The only observed association was between NO_2_ exposure and PD comparing the lowest and highest quartiles.^164^ Other studies have reported an association between PM2.5 exposure and PD,^165^, ^166^ but some did not observe an association.^167^ In a recent study in California applying 10‐year average exposure and a 5‐year lag time, significant associations with carbon monoxide (CO) and PM2.5 were observed.^168^ A cohort study of ~300,000 participants identified from the UK Biobank (UKB) applied four different statistical methods and reported consistent results for an association between PM2.5 and PD for all four methods.^169^

The association with other pollutants has been studied less, with an association reported for NO_2_,^167^ conflicting results for PM10^167^, ^170^ and no association with sulfur dioxide (SO_2_).^171^ A recent Mendelian randomization (MR) study of 456,380 participants in the UKB reported a possible association between genetically estimated NO_2_ exposure and PD.^172^

Ultrafine particles and PM2.5 can trigger inflammatory responses both in the periphery and the central nervous system. After inhalation, PMs can directly interact with alveolar macrophages and airway epithelial cells, leading to local pro‐inflammatory cytokine release and pulmonary inflammation.^173^ This leads to systemic inflammation and oxidative stress. Fine particles in air pollution can directly enter the bloodstream via the alveolar–blood barrier. Chronic systemic inflammation and oxidative stress, together with a potential increase in the permeability of the BBB, may lead to neuroinflammation.^159^, ^174^ In addition to the proposed lung–brain axis route, another entry route might be the direct translocation through olfactory nerve terminals.^175^, ^176^

A few studies have investigated the direct links between air pollution and α‐syn pathology where induction of α‐syn fibrillation and promotion of dopaminergic cell death associated with increased microglia activation was observed *in vitro*.^177^, ^178^ In rodent models, short‐term PM10 exposure resulted in pulmonary and systemic inflammation, while long‐term exposure resulted in motor impairment and dopaminergic cell death in the SN.^178^ Moreover, inhalation of PM2.5 induced microstructural changes in the olfactory bulb and nigrostriatal pathways,^179^ while direct intranasal administration of PM2.5 resulted in an extensive propagation of α‐syn pathology via the olfactory bulb in α‐syn A53T transgenic mice.^177^ In another mouse model, the exposure to nanoparticles after injection of preformed murine α‐syn fibrils failed to significantly affect α‐syn propagation.^180^

Further evidence for an association of air pollution with α‐syn aggregation in humans has been provided by a series of autopsy studies.^181^, ^182^, ^183^, ^184^ The studies investigated α‐syn in the brains of children and young adults living in Metropolitan Mexico City who had been exposed to high concentrations of PM2.5, ultrafine PM, and nanoparticles. They found nanoparticles, α‐syn pathology, and Lewy neurites in the olfactory bulb and SN, along with a disruption of the BBB, increased inflammation, and oxidative stress reactions.^181^, ^182^, ^183^, ^184^ However, these studies also found pathological hallmarks of other neurodegenerative diseases, including AD, indicating again that the potential effects of air pollution are not limited to PD. A link between air pollution and PD is plausible but the available evidence is still limited, and further studies are needed, in particular studies relating to specificity and temporality (Table 3).

**TABLE 3:** Visualization of the association between Parkinson's disease and selected pesticides, trichloroethylene, and air pollutants using the Bradford Hill criteria

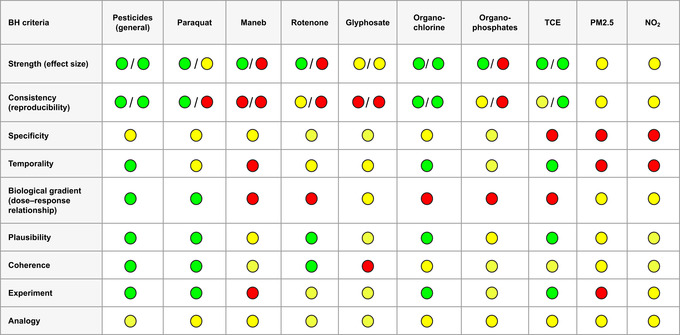										

Case–control studies/cohort studies. Green: good evidence, yellow: some evidence, red: no/lack of evidence.

Abbreviations: BH, Bradford Hill; TCE, trichloroethylene; PM, particulate matter.

## Synthesis of Evidence Quality

We have summarized studies evaluating associations between PD and pesticides, TCE, and air pollutants. Aspects of design may contribute to heterogeneous results in observational studies, including case ascertainment, duration and timing of exposure periods, and exposure assessment methods, as well as assumptions made in the analysis (Box 2). A relatively small proportion of observational studies have looked at specific exposures (ie, specific pesticides and/or co‐exposure) making it difficult to decipher the specific effect of a given exposure on PD risk. For example, the composition of the air pollution mixture, varying levels of different pollutants, and sources of pollution may lead to differences in the toxicity. In environmental epidemiology, the magnitude of exposure misclassification depends heavily on the exposure assessment methods. Other factors contributing to contradictory results are the variations in study design (eg, cohort, case–control, cross‐sectional), case definitions (PD vs. parkinsonism), and case ascertainment (medical record review vs. neurologist assessment vs. self‐report vs. cause of death) are potential sources of bias.^185^

Box 2 **Gaps in evidence for pesticides, trichloroethylene, and air pollution as risk factors for Parkinson's disease**

**Epidemiological Studies**

- *Study design*: Studies need to include lag times and detailed exposure assessment (route, type and measurement, long‐term averaging, cumulative dose, etc.), larger sample sizes, and prospective (cohort) designs. There is a need for accurate Parkinson's disease (PD) diagnosis and a sufficient lag‐time between exposure and outcome in the analysis of risk for PD development considering the duration of the prodromal stage of PD which can occur 20 years before motor symptoms. Additionally, most studies to date have been too reliant on self‐reported exposure data rather than quantitative exposure data collection using wearables.
- *Susceptible populations*: Studies need to be designed to identify susceptible populations (eg, age, sex, occupation) as the exposure might have a different impact in different populations. Similarly, studies of gene–environment interactions (GxE) and co‐exposure need to be done in susceptible populations.
- *Diversity*: More studies from low‐ and middle‐income countries are needed as exposure patterns differ across regions (ie, air pollution mixture differs, and exposure levels are higher, differences in types of pesticides and exposure levels, illegal use of pesticides).
- *Lack of studies*: Few epidemiological studies exist for trichloroethylene (TCE) and air pollution in general.

**Pathophysiology Studies**

- *Doses*: Animal models should better replicate the course and magnitude of exposure to humans, using long‐term exposure to lower doses rather than short‐term application of high doses of toxicants.
- *Route of exposure*: Exposure routes in animal studies should be aligned with those in humans, including simultaneous exposure via different routes. This allows the investigation of the diverse pathways of pathology in humans, including the gut–brain or lung–brain axis.
- *Co‐exposures*: More studies of co‐exposures are needed. Pathophysiological effects on neuroinflammation, oxidative stress, and mitochondrial dysfunction may accumulate in regions or occupations where exposure to different pesticides, air pollution, and/or TCE occur together. Additional studies measuring co‐exposure and temporally relevant toxicants (ie, TCE and other solvents) are also needed.
- *Lack of studies*: More high‐quality pathophysiological studies are needed for comprehensive hazard assessment of exposures discussed in this review where only a few studies exist (ie, maneb), with relevant routes of exposure.

**Policy Change**

- *Transparency in reporting*: Chemical manufacturers and industry should work closely with academic/scientific communities for data sharing, and all relevant safety studies should be reported to regulatory bodies.
- *Regulatory control*: Increased regulatory control, especially in developing countries in which illegal marketing is prominent.
- *Timely discussion*: Concerns from epidemiological or pathophysiological studies should prompt rapid discussion amongst unbiased groups (such as the intergovernmental science–policy panel), without waiting for dedicated meetings.
- *Viable alternatives*: Research should be funded to find efficacious alternatives to harmful chemicals that are cost‐effective and accessible to the end user.

The similar key pathophysiological mechanisms for pesticides, TCE, and certain air pollutants highlight the need for more comprehensive exposure assessment and novel study designs, which can handle complex exposure matrices that contain data on several exposures with similar pathophysiological mechanisms. The evidence from *in vitro*, *in vivo*, and human studies is variable for these environmental factors. Whilst animal models for paraquat and rotenone provide evidence that SN dopaminergic neurons are susceptible to oxidative stress induced by these pesticides, the evidence for other pesticides is weaker.

The animal studies providing evidence that TCE may play a specific role in the pathophysiology of PD are fewer, however, they are consistent. Further investigating the pathophysiological mechanisms of TCE is of high importance in understanding its link to PD. Finally, although certain air pollutants have been linked to neuroinflammation, oxidative stress, and BBB damage, most studies have not aimed to identify mechanisms leading to PD, and the exact molecular pathways are unclear.^158^

To evaluate the contribution of pesticides – in particular, TCE and air pollution, as well as other exposures – further studies are needed, given that important gaps in knowledge remain (Box 2). A multidisciplinary approach is desirable, where epidemiological studies inform pathophysiological studies and vice versa. In addition, clinicians, clinical epidemiologists, environmental epidemiologists, toxicologists, and statisticians must collaborate in the study design phase to minimize the risk of bias.

As a summary of the relationship between PD and the environmental factors highlighted in this review we have designed a table (Table 3) to visually examine the BH criteria for each exposure. Rather than prove causation by means of this table, we hope it more clearly outlines where sufficient evidence exists, and highlights where further research would be helpful.

## Policy Implications

Despite gaps in knowledge, there are important implications for policy, alongside a need for further evidence generation. Much of the evidence has existed for decades, yet policy and legislation have remained largely static with respect to limiting the use of some chemicals. This is despite the growing global socioeconomic burden that PD exerts on healthcare systems worldwide, through medical care, social care, and lost employment.^186^ The authors of this review therefore call for urgent evidence generation that will underpin effective policy change, to limit the use of certain pesticides and volatile compounds, and reduce air pollution, in order to reduce adverse health effects. This action is supported by epidemiologists and PD experts, as well as environmentalists and public health experts, who have long been calling for prompt action on limiting and banning some of the chemicals discussed.^66^, ^187^, ^188^ It is important to lay out the landscape of policy and dissect the root causes of inaction.

At the forefront of policy inaction are economic factors. Pesticides play an essential role in the agricultural economy globally, improving crop yield and reducing food scarcity.^189^ Calling for an immediate, blanket ban is not only premature, but would endanger the livelihoods of farmers worldwide, and disproportionately affect the economies of low‐ and middle‐income countries (LMICs).^190^ It is essential that research is funded to find further safe, non‐toxic replacements for potentially toxic pesticides. Some replacements already exist, and agroecological research has uncovered techniques in integrated weed management that have high efficacy without using paraquat‐containing chemicals or chemically similar alternatives.^190^, ^191^ Greater emphasis should be placed on public education to inform about the potential risks for individuals exposed to pesticides. In line with this goes proper labeling and safety instructions of pesticide products in relation to use and disposal. Additionally, education on hygiene habits and accessibility to proper safety measurements (eg, protective equipment for occupational workers) is essential.

Another reason for policy inaction is deferral of decision making at key roundtable meetings. The Conference of Parties to the Rotterdam Convention (COP) meeting has postponed the decision to upgrade paraquat to a “severely hazardous pesticide formulation” at every meeting since 2011.^192^ This is despite the Rotterdam Convention (2004) listing paraquat as a hazardous pesticide, and global experts' recommendations to upgrade paraquat, since 2011. Once a decision has been deferred multiple times, root cause analyses should be prioritized to try and establish a unanimous decision in either direction; or the threshold for ‘consensus’ decisions should be lowered.

Greater transparency from the companies that manufacture chemicals is needed. A recent exposé in *The Guardian* newspaper (UK) highlighted potential “covert manipulation” of data, and lack of data availability, by the paraquat producer Syngenta, LCC. The company appears to have suppressed important evidence on the links between paraquat and PD.^193^ Recent perspectives have also highlighted poor transparency in the case of glyphosate's EU re‐approval application, alongside neurotoxicity more generally, with key data being withheld.^194^, ^195^ Appropriate policy decisions can never be made if data disclosure is incomplete.

A global approach to regulation is needed. Despite most nations having access to the same body of evidence, there are arbitrary regional differences in quantitative limits on various chemicals.^163^ For example, the UK, EU, and US all have different limits on safe workplace exposure to TCE.^196^ Germany and France have recently recognized “Parkinson's syndrome caused by pesticides” as a new occupational disease,^197^ whereas other countries have not. Policy change would benefit from a centralized, unbiased approach to TCE and pesticide limits, without the influence of competing economic interests from industry. Regional differences in legal limits on air pollutant thresholds exist across the globe. In the case of air pollution, the reasons for regional differences are known to be secondary to regional economic and political interests. However, air pollution benefits from widespread political and non‐governmental organization‐driven exposure, giving it existing priority in policy discussions. This is largely because of the wider (non‐PD) health problems that air pollutants cause, as well as direct environmental consequences,^198^ and the clear overlap with climate change. The intergovernmental science–policy panel, set up in 2022, is one such vehicle through which unbiased policy change can be made.^199^

Globally, 99% of the population is exposed to ambient air pollution exceeding WHO guideline values, leading to 4.5 million premature deaths. Almost 90% of these deaths occur in LMICs.^200^ According to the United Nations environment program, about one‐third of countries (of 194 US states and the EU in 2021) do not have ambient air quality standards embedded in their legislation, with only a fraction of them at least having air quality guidelines or policies.^201^ Furthermore, existing legally binding air quality standards permit higher exposure than recommended by the WHO.^202^ Even in Europe, which has comparable strict air quality legislation, over 80% of the population is exposed to unsafe air pollution levels.^203^ Whilst it is unlikely that PD will be the reason for major changes to legislation, air quality legislation might have beneficial effects on PD incidence.

Taken together, our review of the published literature supports that general exposure to pesticides, and potentially exposure to specific pesticides (eg, paraquat), TCE, and air pollutants, are associated with PD. When considered in isolation, it is difficult to determine whether these associations are causal, in large part because of the decades‐long lag between relevant exposures and the incidence of manifest PD. However, when considered in tandem with evidence from complementary research lines (such as animal models), it is increasingly likely that these associations reflect harmful effects. Despite the methodological issues and remaining gaps in knowledge highlighted, the totality of current evidence provides support for the recent WHO recommendation to lower levels of air pollution and to limit the global use of pesticides and TCE as an entry point to the prevention of PD. This could be done through a tridirectional approach, including funding research into alternative non‐toxic chemicals, better transparency and open dialogue, and a global approach to regulation with centrally agreed limits and public education.

## Financial Disclosures of All Authors (For the Preceding 12 Months)

K.A.B. reports a grant from Parkinson's UK, is a member of the GBA1 Canada initiative (G‐Can) Scientific Advisory Board, and is supported by an employment subcontract with Queen Mary University of London (QMUL) to collaborate on the Aligning Science Across Parkinson's Global Parkinson's Genetics Program (ASAP‐GP2). E.S. reports having received speaker honoraria from Zambon. A.F.S.S. report grants from The Michael J. Fox Foundation, CNPq, and Fapergs. S.K.L.D. currently serves on the editorial board of *Neurology, Frontiers of Neurology*, and *Brain Sciences*; has received fees for speaking at conferences and podcasts from AbbVie; and has received research support from the Parkinson's Foundation (PF‐FBS‐2026) and ZonMW. V.K. sits on the advisory boards of AbbVie and Nordic Infucare AB; has received honoraria from AbbVie, Nordic Infucare, Orion Pharma, Eisai, and Teva; and reports grants from The Finnish Parkinson Foundation, The Finnish Cultural Foundation, The Turku University Foundation, and Turku University Hospital (VTR‐funds). A.M.T. reports grants from Horizon Europe, The Michael J. Fox Foundation, and from Amgen paid through the institution of employment outside of the submitted work. A.J.N. reports grants from Parkinson's UK, Barts Charity, Cure Parkinson's, National Institute for Health and Care Research, Innovate UK, Virginia Keiley benefaction, Solvemed, the Medical College of Saint Bartholomew's Hospital Trust, Alchemab, (ASAP‐GP2) and The Michael J. Fox Foundation, and reports consultancy and personal fees from AstraZeneca, AbbVie, Profile, Bial, Charco Neurotech, Alchemab, Sosei Heptares, Umedeor, and Britannia. A.J.N. also reports having share options in Umedeor and is an Associate Editor for the *Journal of Parkinson's Disease*. A.K., I.K.R., and L.M.C. have no financial disclosures.

## Data Availability

Data sharing is not applicable to this article as no new data were created or analyzed in this study.
